# Supplementary material for: Comparison of geological clusters between influenza and COVID-19 in Thailand with unsupervised clustering analysis
Source: PLoS One. 2024 Jan 22;19(1):e0296888. doi: 10.1371/journal.pone.0296888 (PMC10802941; doi:10.1371/journal.pone.0296888)
Supplement: Attachment — Submitted filename: Response to Reviewer2.pdf [file pone.0296888.s002.pdf]

Reviewer #2: This study represents the use of unsupervised clustering techniques (especially, DBSSCAN) to identify clusters of disease trends in Thailand. Three distinct surveillance datasets, namely the pandemic influenza outbreak, influenza in the endemic stage, and the early stages of COVID-19, were then used as time-series data of the number of infected people in those periods. They revealed similarities between influenza and COVID-19 transmission patterns during the pandemic phases, including the number of provinces in the clusters and spatial distribution patterns. A pair of provinces with highly similar spreading patterns during both the pandemic stages of influenza and COVID-19 was also reported. It was a great analysis to study the patterns of the endemic in Thailand. However, there are some concerns and limitations that need addressing:

1. The authors should explore the statistics of the data used and perform necessary preprocessing before applying clustering.

- I decided to revert to use original monthly data and added explanation about preprocessing data in line 167-169, 172-174, 180-183, and 201-212.

2. The choice of weekly data for analysis needs clarification, as it might result in repetitive values, making it similar to using monthly data. Why would the authors perform the analysis for weekly data?

- I have reverted to using the original monthly data to prevent minor errors. The results do not show significant deviations from the previous experiment. I have already incorporated these revisions into the updated results.

3. The time-series data for all provinces should be visually presented to observe any common distribution patterns among them. The plot of these can show us the outliers and common groups of provinces. These can also be applied to all three data sets.

- I aim to visualize the surveillance data from our three datasets. Regrettably, when I attempted to plot data from all 77 provinces together, it resulted in a cluttered visualization. Consequently, I've decided to create more visually appealing representations by focusing on specific clusters and comparing them with major provinces in figures 8-10.

4. The authors should explain how they selected the number of clusters in the DBSCAN algorithm and explore different parameters to improve clustering results. The reasons for choosing DBSCAN as the clustering algorithm and determining optimal parameters (e.g., epsilon and minimum points) should be elaborated.

- I've added more explanation about DBSCAN and our fine-tuning parameters in line 215-255.

5. The authors should address the situations or policies that could have contributed to the resulting clusters, such as the behavior of people, travel patterns, or government policies. Practical implications for policymakers and public health officials need to be discussed, explaining how this knowledge can inform decision-making and response strategies during future pandemics or endemic phases. What are the situations or policies behind the resulting clusters? These should be clearly addressed. It might be because of the behavior of the Thai people or because workers always come to Bangkok and go back to their homes during long holidays or a knock-down policy.

- I've added information related to public health policies in line 68-81 and case study in Mexico and Singapore in line 86-92.

6. The authors should compare their findings with previous studies on similar topics to validate their results and strengthen their conclusions. Some other clustering approaches may provide different clustering results.

- I have attempted to identify similar clusters for H1N1 and COVID-19 in Thailand but haven't found a match yet. Before choosing DBSCAN as a clustering method, I experimented with other techniques such as K-means and Self-Organizing Map (SOM), but unfortunately, they did not yield significant results.

7. Limitations, including inherent biases in clustering analysis and potential missed aspects of disease transmission, should be acknowledged.

- I've added technical limitation in 247-250.

8. All figures should be properly labeled with axis titles, and higher-resolution figures should be provided to improve clarity. It may be not necessary to have Figure 1 in the circumstance of the analysis. Only report the number in the text would be enough.

- I've added the axis labels for all figures and recheck the resolution of figures.

9. There is a lack of discussion part that the author can discuss the methods, results, practical implications, and limitation of their study.

- I've added more discussion and explanation in method part and result part.
