# Supplementary material for: Comparison of geological clusters between influenza and COVID-19 in Thailand with unsupervised clustering analysis
Source: PLoS One. 2024 Jan 22;19(1):e0296888. doi: 10.1371/journal.pone.0296888 (PMC10802941; doi:10.1371/journal.pone.0296888)
Supplement: Attachment — Submitted filename: renamed_291bf.docx [file pone.0296888.s001.docx]

**Revision of the article «Comparison of Geological Clusters between Influenza and COVID-19 in Thailand with Unsupervised Clustering Analysis»**

The article aimed to investigate the transmission patterns of influenza and COVID-19 in Thailand using unsupervised clustering analysis. The findings of this study have important implications for public health management and preparedness for future pandemics or endemic phases. I have outlined my comments and suggestions below to improve the manuscript for potential publication:

**General Comments:**

1. Abstract:

The abstract provides a concise summary of the study. However, it would be beneficial to include specific findings or key results from the analysis in the abstract to make it more informative.

2. Introduction:

The introduction provides a good background of the study, but it can be further improved by including more recent references related to COVID-19 and influenza, especially since the pandemic has likely progressed since the time of your knowledge cutoff. Additionally, please ensure that all references cited in the introduction are appropriately cited in the main text.

3. Methods:

Provide more details on the data collection process from the Department of Disease Control (DDC), including any potential limitations or biases in the datasets used.

Clarify the rationale for averaging the influenza surveillance data into a daily time-series format before converting it into a weekly time-series format.

Explain how you determined the optimal values for ε (epsilon) and MinPts parameters for the DBSCAN algorithm.

4. Results:

Ensure that all tables and figures are properly referenced in the main text. Additionally, consider providing more descriptive captions for figures to enhance their understanding.

Provide more detailed insights and interpretations of the clustering analysis results. Discuss the significance of the observed variations in the spread patterns of influenza and COVID-19 among different provinces in Thailand.

5. Discussion:

It is crucial to include a dedicated discussion section to interpret the study's findings in light of the existing literature. Discuss the implications of the similarities and differences in the clustering patterns of influenza and COVID-19 during the pandemic and endemic phases. Relate your findings to the broader context of public health management and preparedness for future pandemics.

6. Language and Clarity:

Proofread the manuscript thoroughly for language errors and clarity. Consider revising some sentences to improve readability.

**Specific Comments**

**Abstact:**

While the objective is implied, it could be explicitly stated at the beginning of the abstract. For example: "The objective of this study was to identify and compare the transmission patterns of influenza and COVID-19 in Thailand."

Provide more specific details about the findings. Instead of using terms like "similarities" and "different patterns," mention the specific aspects that were similar and different between influenza and COVID-19 in the pandemic and endemic stages.

Include a sentence at the end of the abstract that highlights the significance of the study's findings and how they can contribute to public health management.

**Introduction**

1. Begin the introduction by providing a concise background on the worldwide outbreak of the coronavirus disease (COVID-19) pandemic in early 2020 and its significant impact on public health. Include statistics or references to highlight the scale of the pandemic and its global consequences.
2. Emphasize the importance of understanding the transmission patterns of respiratory pathogens, such as influenza, in the context of the COVID-19 pandemic. Briefly discuss the potential implications of changes in transmission patterns for public health management and preparedness for future pandemics.
3. Provide a more detailed and chronological account of the 2009 influenza pandemic (H1N1) to establish its significance as the first human influenza pandemic in the 21st century. Include the number of countries affected, confirmed cases, and fatalities to underscore its impact on global health.
4. Add more information on the public health measures implemented during the COVID-19 pandemic and their impact on mitigating the spread of both influenza and COVID-19. Include references to studies or official reports that support the claim of lower influenza cases and associated hospitalizations during the pandemic.
5. Clarify the connection between COVID-19 and influenza as both being respiratory infections that initially lacked vaccination-based immunity in the population.
6. While the general objective of identifying spread patterns in specific regions and provinces is mentioned, it could be more explicitly stated at the end of the introduction.
7. Clearly state that your research aims to provide quantitative and spatial information to aid public health management in preparing for future pandemics or transitioning into an endemic phase. Highlight the practical implications of your findings for public health strategies and decision-making.

Example of reference you may used in the text:

Kumar, S., Havens, P., Chusid, M., Willoughby, R., Simpson, P., & Henrickson, K. (2010). Clinical and Epidemiologic Characteristics of Children Hospitalized With 2009 Pandemic H1N1 Influenza A Infection. The Pediatric Infectious Disease Journal, 29, 591-594. <https://doi.org/10.1097/INF.0b013e3181d73e32.>

Yousfi, N., Bragazzi, N. L., Briki, W., Zmijewski, P., & Chamari, K. (2020). The COVID-19 pandemic: how to maintain a healthy immune system during the lockdown–a multidisciplinary approach with special focus on athletes. Biology of sport, 37(3), 211-216.

González-Sanguino, C., Ausín, B., Castellanos, M., Sáiz, J., López-Gómez, A., Ugidos, C., & Muñoz, M. (2020). Mental health consequences during the initial stage of the 2020 Coronavirus pandemic (COVID-19) in Spain. Brain, Behavior, and Immunity, 87, 172 - 176. <https://doi.org/10.1016/j.bbi.2020.05.040.>

Soo, R., Chiew, C., Ma, S., Pung, R., & Lee, V. (2020). Decreased Influenza Incidence under COVID-19 Control Measures, Singapore. Emerging Infectious Diseases, 26, 1933 - 1935. <https://doi.org/10.3201/eid2608.201229.>

Arellanos-Soto, D., Padilla-Rivas, G., Ramos-Jiménez, J., Galán-Huerta, K., Lozano-Sepúlveda, S., Martínez-Acuña, N., Treviño-Garza, C., Montes-de-Oca-Luna, R., de-la-O-Cavazos, M., & Rivas-Estilla, A. (2021). Decline in influenza cases in Mexico after the implementation of public health measures for COVID-19. Scientific Reports, 11. <https://doi.org/10.1038/s41598-021-90329-w.>

**Methods**

1. Provide more details about the data sources from the DDC, such as the methods used for data collection, the geographic coverage (i.e., data from each province), and any potential limitations or biases in the data that should be considered in the analysis.
2. In the section about the International Air Transport Association (IATA) datasets, specify the time period covered by the air travel data used to identify the countries with the highest frequency of air travel to and from Thailand. Explain how this data will be integrated into the analysis of transmission patterns.
3. When converting the influenza surveillance data from monthly to daily and then to weekly time-series formats, provide a clear rationale for choosing these time intervals. Explain how this data transformation process affects the subsequent analysis.
4. Describe the normalization process used to convert the total number of influenza cases for each province into the number of cases per 100,000 people. Clarify why this normalization method was chosen and how it enables meaningful comparisons between provinces with different population sizes.
5. Provide a more detailed explanation of the Density Based Spatial Clustering of Applications with Noise (DBSCAN) algorithm. Explain the concepts of neighborhood and connectivity used to determine clusters and how the parameters (ε and MinPts) were selected or optimized for the for the DBSCAN algorithm.
6. Clarify how the three categories of data points (core, border, and outlier) are identified and utilized in the DBSCAN clustering analysis. Elaborate on how these categories contribute to identifying dense regions and outliers in the data.
7. Mention any potential limitations or challenges associated with using the DBSCAN algorithm for clustering time-series data. Discuss how these challenges were addressed to ensure the reliability and validity of the clustering results.

**Results**

1. Clearly state the main findings of the clustering analysis in a concise manner at the beginning of the "Results" section.
2. Provide a summary of the number of clusters identified and the provinces included in each cluster for both influenza and COVID-19 datasets.
3. When discussing the results in the text, highlight the most significant observations from Table 2. Emphasize the variations in transmission patterns between the different stages of influenza (pandemic vs. endemic) and COVID-19, and explain the implications of these differences on public health management and future pandemic preparedness.
4. Provide a more detailed interpretation of the clustering maps shown in Figs 2-4. Describe the geographic distribution of clusters in Thailand, and discuss any regional patterns or trends observed.
5. Provide a clear explanation of the fluctuations or trends observed in the infection rates of influenza and COVID-19 in Roi Et and Maha Sarakham provinces.
6. Discuss the practical implications of the observed differences in infection trends among the major provinces. Address how these findings can inform targeted public health interventions and resource allocation in different regions of Thailand
